# Generation and Standardized, Systemic Phenotypic Analysis of *Pou3f3^L423P^* Mutant Mice

**DOI:** 10.1371/journal.pone.0150472

**Published:** 2016-03-22

**Authors:** Sudhir Kumar, Birgit Rathkolb, Elisabeth Kemter, Sibylle Sabrautzki, Dian Michel, Thure Adler, Lore Becker, Johannes Beckers, Dirk H. Busch, Lillian Garrett, Wolfgang Hans, Sabine M. Hölter, Marion Horsch, Martin Klingenspor, Thomas Klopstock, Ildikó Rácz, Jan Rozman, Ingrid Liliana Vargas Panesso, Alexandra Vernaleken, Andreas Zimmer, Helmut Fuchs, Valérie Gailus-Durner, Martin Hrabě de Angelis, Eckhard Wolf, Bernhard Aigner

**Affiliations:** 1 Chair for Molecular Animal Breeding and Biotechnology, and Laboratory for Functional Genome Analysis (LAFUGA), Gene Center, LMU Munich, 81377, Munich, Germany; 2 German Mouse Clinic, Institute of Experimental Genetics, Helmholtz Zentrum München, German Research Center for Environmental Health, 85764, Neuherberg, Germany; 3 Member of German Center for Diabetes Research (DZD), 85764, Neuherberg, Germany; 4 Institute of Medical Microbiology, Immunology, and Hygiene, TU Munich, 81675, Munich, Germany; 5 Chair of Experimental Genetics, Center of Life and Food Sciences Weihenstephan, TU Munich, 85350, Freising-Weihenstephan, Germany; 6 Institute of Developmental Genetics, Helmholtz Zentrum München, German Research Center for Environmental Health, 85764, Neuherberg, Germany; 7 Molecular Nutritional Medicine, Else Kröner-Fresenius Center, TU Munich, 85350, Freising-Weihenstephan, Germany; 8 Department of Neurology, Friedrich-Baur-Institut, LMU Munich, 80336, Munich, Germany; 9 German Center for Vertigo and Balance Disorders, University Hospital Munich, Campus Grosshadern, 81377, Munich, Germany; 10 Laboratory of Molecular Neurobiology, Department of Psychiatry, University of Bonn, 53105, Bonn, Germany; Ohio State University Comprehensive Cancer Center, UNITED STATES

## Abstract

Increased levels of blood plasma urea were used as phenotypic parameter for establishing novel mouse models for kidney diseases on the genetic background of C3H inbred mice in the phenotype-driven Munich ENU mouse mutagenesis project. The phenotypically recessive mutant line HST011 was established and further analyzed. The causative mutation was detected in the POU domain, class 3 transcription factor 3 (*Pou3f3*) gene, which leads to the amino acid exchange *Pou3f3*^*L423P*^ thereby affecting the conserved homeobox domain of the protein. *Pou3f3* homozygous knockout mice are published and show perinatal death. Line *Pou3f3*^*L423P*^ is a viable mouse model harboring a homozygous *Pou3f3* mutation. Standardized, systemic phenotypic analysis of homozygous mutants was carried out in the German Mouse Clinic. Main phenotypic changes were low body weight and a state of low energy stores, kidney dysfunction and secondary effects thereof including low bone mineralization, multiple behavioral and neurological defects including locomotor, vestibular, auditory and nociceptive impairments, as well as multiple subtle changes in immunological parameters. Genome-wide transcriptome profiling analysis of kidney and brain of *Pou3f3*^*L423P*^ homozygous mutants identified significantly regulated genes as compared to wild-type controls.

## Introduction

Biomedical research with mice as animal models includes the search for and the analysis of alleles that predispose for or protect against specific diseases. A strategy for the search of novel disease-related alleles consists of the random chemical mutagenesis of a large number of animals followed by the systematic screening for clinically relevant disease phenotypes. The alkylating agent *N*-ethyl-*N*-nitrosourea (ENU) is mutagenic for premeiotic spermatogonial stem cells and allows the production of a large number of randomly mutagenized offspring from treated males. ENU predominantly induces point mutations [1]. In the phenotype-driven Munich ENU mouse mutagenesis project using C3HeB/FeJ (C3H) inbred mice as genetic background, a standardized screening profile of clinical chemical blood plasma parameters was established for the analysis of the offspring of the mutagenized mice in order to detect phenotypic variants [2,3]. Several mutant lines were established showing increased plasma urea levels as a parameter that is indicative of kidney diseases [4].

*Pou3f3* (POU domain, class 3 transcription factor 3, also denominated as *Brn1*) is an intronless gene and is GC rich (74%) throughout the coding region [5,6]. It encodes a 497 amino acid protein that carries a common DNA binding motif called POU domain (aa 403–462). The amino acid sequence of POU3F3 is highly conserved in mouse and human thereby showing no differences in the POU domain but two amino acid exchanges as well as two sites harbouring glycine stretches with different length in the rest of the sequence (http://www.ensembl.org). POU transcription factors regulate a variety of developmental processes. POU3F3 plays a role in neuronal development and is expressed in the developing neocortex, both in the late precursor cells and in the migrating neurons (http://www.uniprot.org). The MGI database (http://www.informatics.jax.org) harbouring knockout as well as mutant mouse alleles includes two complete POU3F3 knockouts. *Pou3f3* homozygous knockout mice show neonatal mortality. One-day-old homozygous knockout mice have increased plasma urea and potassium levels with renal hypoplasia and show developmental defects in the forebrain and the loop of Henle [7,8].

The ENU mutagenesis-derived recessive mutant mouse line HST011 showing increased plasma urea levels was analyzed for the causative mutation. After the identification of the causative mutation in *Pou3f3*, a standardized, systemic phenotypic analysis of *Pou3f3*^*L423P*^ homozygous mutant mice was carried out in the German Mouse Clinic (http://www.mouseclinic.de) to examine organ systems and/or pathways that may be affected by the *Pou3f3* mutation as primary or secondary effects.

## Materials and Methods

### Animals, Linkage Analysis, and Detection of the Causative Mutation

The recessive mutant line HST011 (= UREHR2) was established in the clinical chemical screen of the phenotype-based Munich ENU mouse mutagenesis project [9] on the C3HeB/FeJ (C3H) inbred genetic background by showing increased plasma urea values at the age of three months (cut-off level: 70 mg/dl = 11.7 mmol/l). Mouse husbandry, breeding, linkage analysis, and genome-wide mapping were performed as described previously [4]. All mice had free access to drinking water and a standard rodent diet (V1124; Ssniff, Soest, Germany) *ad libitum*.

For linkage analysis of the causative mutation, automated DNA extraction from the lysates was performed using the AGOWA Mag Maxi DNA Isolation Kit (AGOWA, Berlin, Germany). A genome-wide, evenly distributed mapping panel consisting of 158 single nucleotide polymorphism (SNP) markers was applied. The markers used are available upon request. Genotyping using this panel was performed by MassExtend, a MALDI-TOF high-throughput genotyping system supplied by Sequenom (San Diego, CA, USA). Additional fine mapping was performed using further SNP and microsatellite markers. Chromosomal positions of markers and genes are according to the GRCm38 mouse assembly (http://www.ensembl.org). All genes which are located in the identified defined chromosomal region were analyzed for published data about their wild-type and mutant function with respect to their potential impact on renal function and renal diseases.

### Immunohistochemical Analysis

For immunohistochemical analysis, kidneys were fixed with 4% paraformaldehyde in PBS (pH 7.4) via orthograde vascular perfusion, postfixed by immersion in the same fixative for one day and embedded in paraffin. 3 μm thick paraffin sections were dewaxed and rehydrated, and heat induced antigen retrieval using Citrate buffer (pH 6.0) was performed. Immunohistochemistry was performed with a polyclonal guinea pig anti-POU3F3 antiserum (generous gift from Michael Wegner) [10] and HRP-labeled rabbit anti-guinea pig antibody (Dako, Hamburg, Germany). Immunoreactivity was visualized using 3,3-diaminobenzidine tetrahydrochloride dihydrate (DAB) (brown color) and nuclear counterstaining was done with haemalum (blue color).

### Phenotypic Analysis in the German Mouse Clinic

Maintenance of the recessive mutant mouse line *Pou3f3*^*L423P*^ comprised the repeated backcross to C3H wild-type mice for more than ten generations leading to the subsequent loss of essentially all non-causative ENU mutations that were not linked to the *Pou3f3* mutation. The comprehensive phenotypic analysis was carried out in the German Mouse Clinic at the Helmholtz Zentrum München by using standardized examination protocols (http://www.mouseclinic.de). The analysis covers several hundred parameters in the areas of allergy, behavior, cardiovascular analysis, clinical chemistry, dysmorphology including bone and cartilage, energy metabolism, eye analysis and vision, immunology, lung function, molecular phenotyping, neurology, nociception, steroid analysis, and pathology. The complete protocols of the examinations are described under http://www.mouseclinic.de [11–13].

*Pou3f3*^*L423P*^ homozygous mutant mice were analyzed with an age of 8–24 weeks. 27 homozygous mutants and 32 wild-type control littermates were used for phenotypic analyses. The number of animals analyzed was 13–16 animals per sex and genotype (except of otherwise stated in the text of the respective Results section).

Mouse husbandry was done under a continuously controlled specific pathogen free (SPF) hygiene standard according to the FELASA recommendations [14] (http://www.felasa.eu). All tests were carried out under the approval of the responsible animal welfare authority (Regierung von Oberbayern).

Data are shown as mean ± standard deviation. If not otherwise stated, data were analyzed using R, a language and environment for statistical computing. Tests for genotype effects were made by using *t*-test, Wilcoxon rank sum test, linear models, or ANOVA depending on the assumed distribution of the parameter and the questions addressed to the data. Significant differences are indicated for *P* < 0.05, 0.01, and 0.001.

## Results

### Generation of Line *Pou3f3*^*L423P*^ and Identification of the Causative Mutation

The ENU mutagenesis-derived, recessive mutant line HST011 with the G1 male founder ID 20033899 was established on the C3H inbred genetic background by showing increased plasma urea values at the age of three months (cut-off level: 70 mg/dl = 11.7 mmol/l) in homozygous mutant mice. Homozygous mutants of both sexes were viable and fertile. They exhibited a smaller size but no other grossly apparent phenotype compared to wild-type controls.

**Fig 1:**
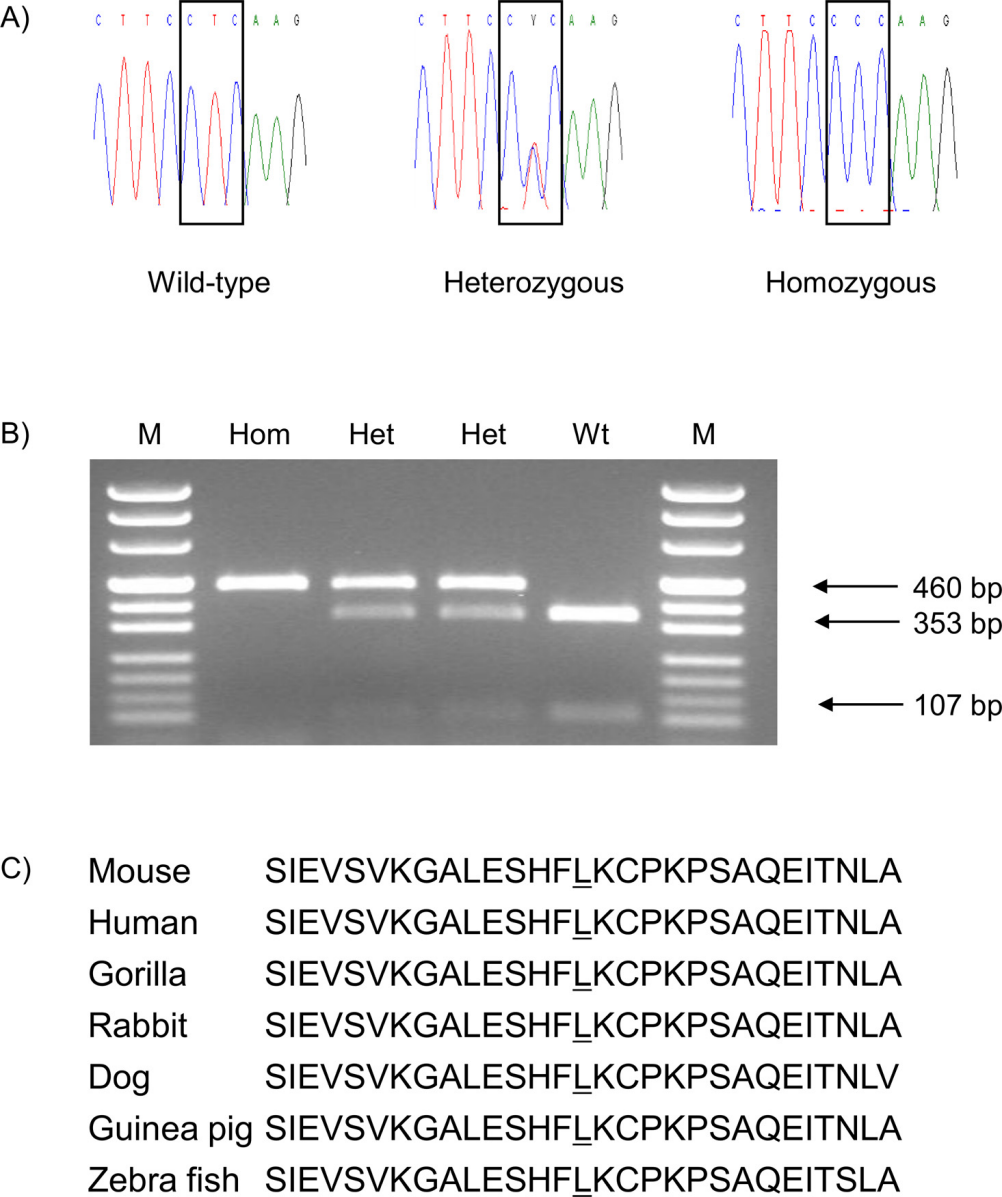
Analysis of *Pou3f3* in wild-type and *Pou3f3*^*L423P*^ mutant mice. (A) Electropherogram of the *Pou3f3*^*L423P*^ mutation. The box shows the T→C point mutation at nt 1268 (ENSMUST00000054883) from the wild-type codon leucine (L) to the mutant codon proline (P) at amino acid position 423. (B) Genotyping of mice by allele-specific PCR-RFLP reaction. *Sml*I restriction digest of the 460 bp PCR product results in 353 bp and 107 bp fragments of the wild-type allele. Hom, *Pou3f3*^*L423P*^ homozygous mutant; Het, *Pou3f3*^*L423P*^ heterozygous mutant; Wt, wild-type; M, pUC Mix 8 marker, MBI Fermentas. (C) Partial protein sequence alignment of the homeobox domain of murine POU3F3 with other species. The underlined amino acid residue shows the position of the *Pou3f3*^*L423P*^ mutation.

Genome-wide linkage analysis of the causative mutation was carried out with phenotypically homozygous mutant G2 animals derived from the mating of phenotypically homozygous mutant animals on the inbred C3H genetic background with BALB/c inbred mice and the subsequent intercross of the phenotypically inconspicuous heterozygous mutant G1 mice. Using a panel of 116 genome-wide polymorphic markers, the mutant phenotype was mapped to a single locus on MMU1. Further fine mapping showed the highest linkage for the polymorphic marker D1Mit212 (40.0 Mb) (http://www.ensembl.org). The candidate gene *Pou3f3* was selected for the sequence analysis, as it is located at 42.7 Mb on chromosome 1, and a published mutant phenotype has been described with increased plasma urea and potassium levels associated with renal hypoplasia (http://www.informatics.jax.org). *Pou3f3* consists of a single exon coding for a 497 aa polypeptide. Sequence analysis of *Pou3f3* revealed a T→C point mutation at nt 1268 (ENSMUST00000054883) which leads to the amino acid exchange from leucine to proline at aa position 423. Therefore, the name of line HST011 was designated as *Pou3f3*^*L423P*^. The mutation affects the highly conserved DNA binding domain of the protein including aa 403–462. Allelic differentiation of the *Pou3f3*^*L423P*^ mutation was carried out by PCR-RFLP as the point mutation abolished the restriction site for the enzyme *Sml*I (Fig 1).

**Fig 2:**
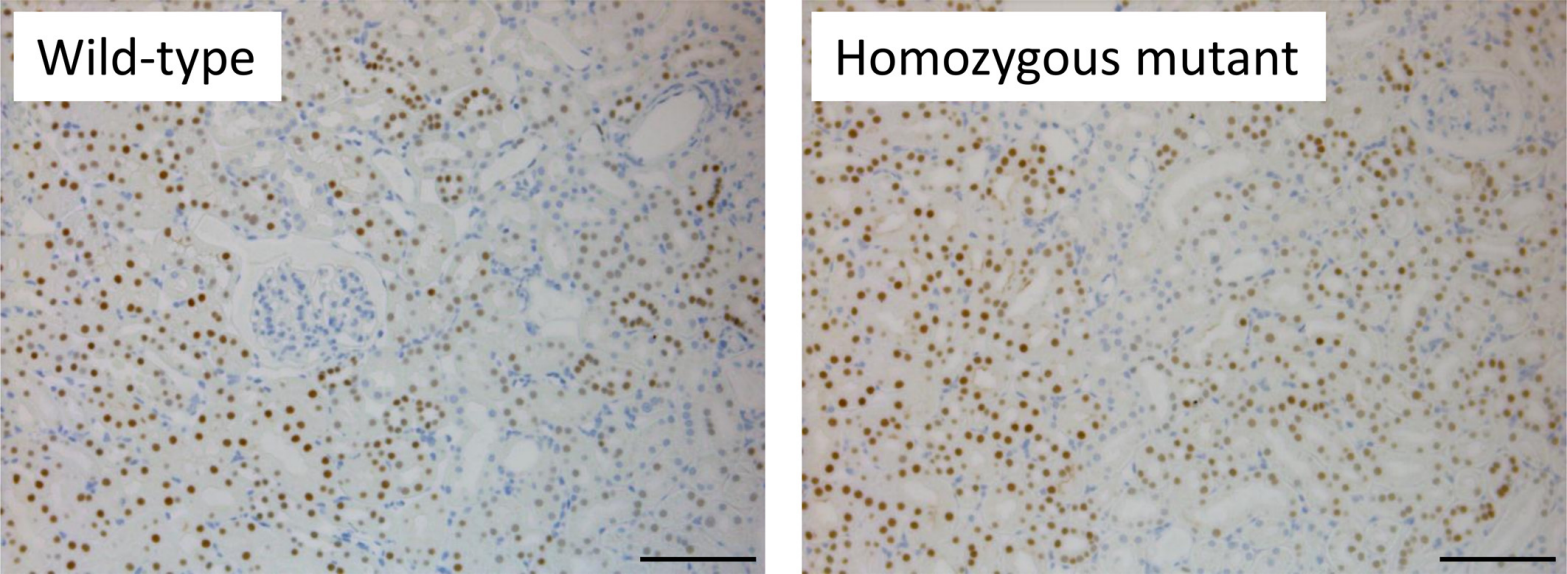
Immunohistochemical localization of POU3F3 in kidneys. POU3F3 was detected in nuclei of numerous but not all tubule segments with weak up to strong staining intensity in a wild-type kidney. The immunostaining pattern and staining intensity of POU3F3 in the kidney of a *Pou3f3*^*L423P*^ homozygous mutant mouse was similar to the findings observed in the wild-type control kidney. DAB (brown color); nuclear staining: haemalum (blue color). Age of mice analyzed: 22 months. Bars represent 100 μm.

To evaluate the impact of the amino acid changing mutation POU3F3^L423P^ on cellular localization and abundance, immunohistochemical localization of POU3F3 in kidneys was performed (Fig 2). In wild-type controls, the transcription factor POU3F3 was immunostained in nuclei of numerous but not all tubule profiles in weak up to strong staining intensity. The kidneys of *Pou3f3*^*L423P*^ homozygous mutant mice exhibited similar staining pattern and staining intensity of POU3F3 compared to wild-type control kidneys.

### Phenotypic Analysis in the German Mouse Clinic

Phenotypic analysis of mutant mice in the German Mouse Clinic includes the aim to collect and deliver by free access systemic phenome data of a high number of mutant mouse lines in a standardized manner. The complete phenotype reports will be deposited online (http://146.107.6.187/phenomap/jsp/annotation/public/phenomap.jsf). *Pou3f3*^*L423P*^ homozygous mutant mice were analyzed under the preliminary line name HST011. Compared to wild-type controls, *Pou3f3*^*L423P*^ heterozygous mutant mice showed no differences in the guiding primary symptoms of plasma urea value and relative kidney weight. Thus, *Pou3f3*^*L423P*^ homozygous mutant mice were examined in the German Mouse Clinic with wild-type littermates as controls (S1 Table).

### Behavioral and Neurological Analysis

*Pou3f3*^*L423P*^ homozygous mutant mice were tested at the age of eight weeks in the open field as a test of spontaneous unconditioned reactions to a novel environment allowing an evaluation of exploratory drive, reactivity to novelty and emotionality. Compared to controls, mutant mice generally showed an increased locomotor activity in this environment with increased distance moved forward and increased average speed of movement. They spent more time in the aversive zone of the center and less time in the periphery, however, this may be secondary due to their increased locomotor activity. In addition, female mutant mice exhibited a decreased rearing activity (Table 1) that is likely due to the balance and vestibular deficits described below.

**Table 1:** Open field behavioral analysis of line *Pou3f3^L423P^*.

	Males		Females		Genotype: *P*-value
Parameter	Homozygous mutant	Wild-type	Homozygous mutant	Wild-type	
Distance traveled–total (cm)	11382 ± 5683	8013 ± 2754	12443 ± 5374	9987 ± 3127	a
Number of rearings—total	6.7 ± 11.0	16.4 ± 23.9	2.3 ± 6.6	47.9 ± 45.3	c
Percent distance in the center—total	18.3 ± 13.0	5.1 ± 5.7	20.3 ± 11.1	9.9 ± 7.0	c
Percent time spent in the center—total	15.0 ± 10.9	3.5 ± 3.8	16.7 ± 9.6	7.0 ± 4.9	c
Whole arena—resting time (sec)	11.1 ± 15.1	24.6 ± 33.8	3.3 ± 4.9	68.5 ± 62.6	c
Whole arena—average speed (cm/sec)	9.6 ± 4.9	6.9 ± 2.6	10.4 ± 4.5	8.9 ± 3.1	a
Latency to enter in the center (sec)	37 ± 59	203 ± 347	89 ± 133	115 ± 129	
Number of entries in the center	131 ± 123	26 ± 30	142 ± 92	54 ± 38	c

8-week-old mice were tested in the open field for 20 min. No. per genotype and sex: n = 12–16. Data are presented as mean ± standard deviation. Significance vs. wild-type controls: a, *P* < 0.05; c, *P* < 0.001.

Body weights of mutant mice were lower than that of the controls. Already at eight weeks of age, the mean body weight reduction was 19% in male mutants and 24% in female mutants. Significantly altered parameters of the SHIRPA analysis (at 8 weeks) were presence of lacrimation, presence of pelvic elevation during walking, and especially mutant mice showed trunk curl upon tail suspension and an impaired contact righting reflex when the mice were turned on their back. The other SHIRPA parameters (locomotor activity, body position, tremor, palpebral closure, defecation and urination during observation, transfer arousal, gait, tail elevation, startle response, touch escape, positional passivity, limb grasping, pinna reflex, corneal reflex, evidence of biting and vocalization in and above the arena) were without significant alterations (Table 2).

**Table 2:** Neurology analysis by modified SHIRPA of line *Pou3f3*^*L423P*^.

	Males		Females		Genotype: *P*-value
Parameter	Homozygous mutant	Wild-type	Homozygous mutant	Wild-type	
Body weight (g)	22.4 ± 1.7	27.7 ± 2.2	18.9 ± 1.8	24.9 ± 2.8	c
Locomotor activity (floor squares crossed)	11.1 ± 10.2	7.4 ± 5.8	10.8 ± 7.9	16.4 ± 7.1	
Lacrimation	5 of 14	0 of 16	6 of 12	0 of 16	c
Pelvic elevation (more than 5)	4 of 14	0 of 16	3 of 12	0 of 16	b
Tail elevation: horizontal extension	4 of 14	0 of 16	5 of 12	4 of 16	0.06
Trunk curl: present	8 of 14	3 of 16	9 of 12	4 of 16	b
Urination: absent	12 of 14	7 of 16	6 of 12	7 of 16	0.07
Contact righting reflex: absent	11 of 14	2 of 16	8 of 12	1 of 16	c

8-week-old mice were tested. No. per genotype and sex: n = 12–16. Data are presented as mean ± standard deviation (body weight, locomotor activity) and as exact numbers. Significance vs. wild-type controls: b, *P* < 0.01; c, *P* < 0.001.

Subsequent measurement of the grip strength (g) for two paws and four paws to evaluate muscle performance revealed decreased values in the mutants (mean force for 2 paws: 101 ± 19 vs. 120 ± 20 in males, 84 ± 16 vs. 119 ± 16 in females (*P* = 0.09); mean force for 4 paws: 184 ± 28 vs. 207 ± 17 in males, 152 ± 8 vs. 198 ± 16 in females (*P* = 0.03); n = 12–16 per genotype and sex) which was attributed mainly to the decreased body weight.

Evaluation of motor coordination and balance in three consecutive trials on the accelerating rotarod at the age of 9 weeks revealed a tendency to increased latencies (sec) on the rod in the light-weight mutants (127 ± 53 vs. 103 ± 35 in males, 113 ± 55 vs. 97 ± 32 in females (*P* = 0.89); n = 12–16 per genotype and sex) and significantly increased passive rotations (45 in three trials of 27 mutants vs. 35 in three trials of 32 controls (*P* = 0.02)). The usual improvement in the performance of the task over the three trials was observed in both genotypes. Since the mutants showed movement deficits hinting towards coordination or balance deficits, additional tests especially sensitive to balance impairments were carried out with the same group of mice at the age of 19 weeks. Balance beam, beam ladder, swim ability as well as gait analysis were performed (n = 10–16 per sex and genotype) with alterations detected in all tests (Table 3).

**Table 3:** Additional locomotor analysis requiring balance maintenance of line *Pou3f3*^*L423P*^.

		Males		Females		Genotype: *P*-value
Test	Parameter	Homozygous mutant	Wild-type	Homozygous mutant	Wild-type	
Balance beam	Square, Ø 20 mm (sec)	28.8 ± 8.4	14.0 ± 7.7	24.5 ± 8.8	10.8 ± 4.8	c
	Square, Ø 12 mm (sec)	25.6 ± 8.5	13.5 ± 7.7	24.0 ± 12.2	12.1 ± 5.5	c
	Round, Ø 22 mm (sec)	22.2 ± 8.2	10.6 ± 5.3	18.6 ± 6.2	9.7 ± 5.4	c
	Round, Ø 15 mm (sec)	23.1 ± 8.4	14.6 ± 7.5	21.3 ± 9.7	15.0 ± 7.7	b
Beam ladder	Time (sec)	22.6 ± 4.4	17.8 ± 6.1	23.1 ± 3.8	17.1 ± 4.4	c
	Fore paw slips (n)	0.5 ± 0.5	0.3 ± 0.5	0.4 ± 0.4	0.2 ± 0.2	a
	Hind paw slips (n)	6.6 ± 2.3	3.4 ± 1.5	5.1 ± 1.8	2.4 ± 1.7	c
	Stops (n)	4.2 ± 1.1	3.3 ± 1.5	4.1 ± 0.9	2.9 ± 1.0	b
Swim ability	Normal (%)	46	100	10	93	c
	Irregular (%)	54	0	90	7	c

Analysis was done at the age of 19 weeks. No. per genotype and sex: n = 10–16. Data are presented as mean ± standard deviation (balance beam, beam ladder). For the swim ability test, the percentages of the mice are shown. Significance vs. wild-type controls: a, *P* < 0.05; b, *P* < 0.01; c, *P* < 0.001.

**Fig 3:**
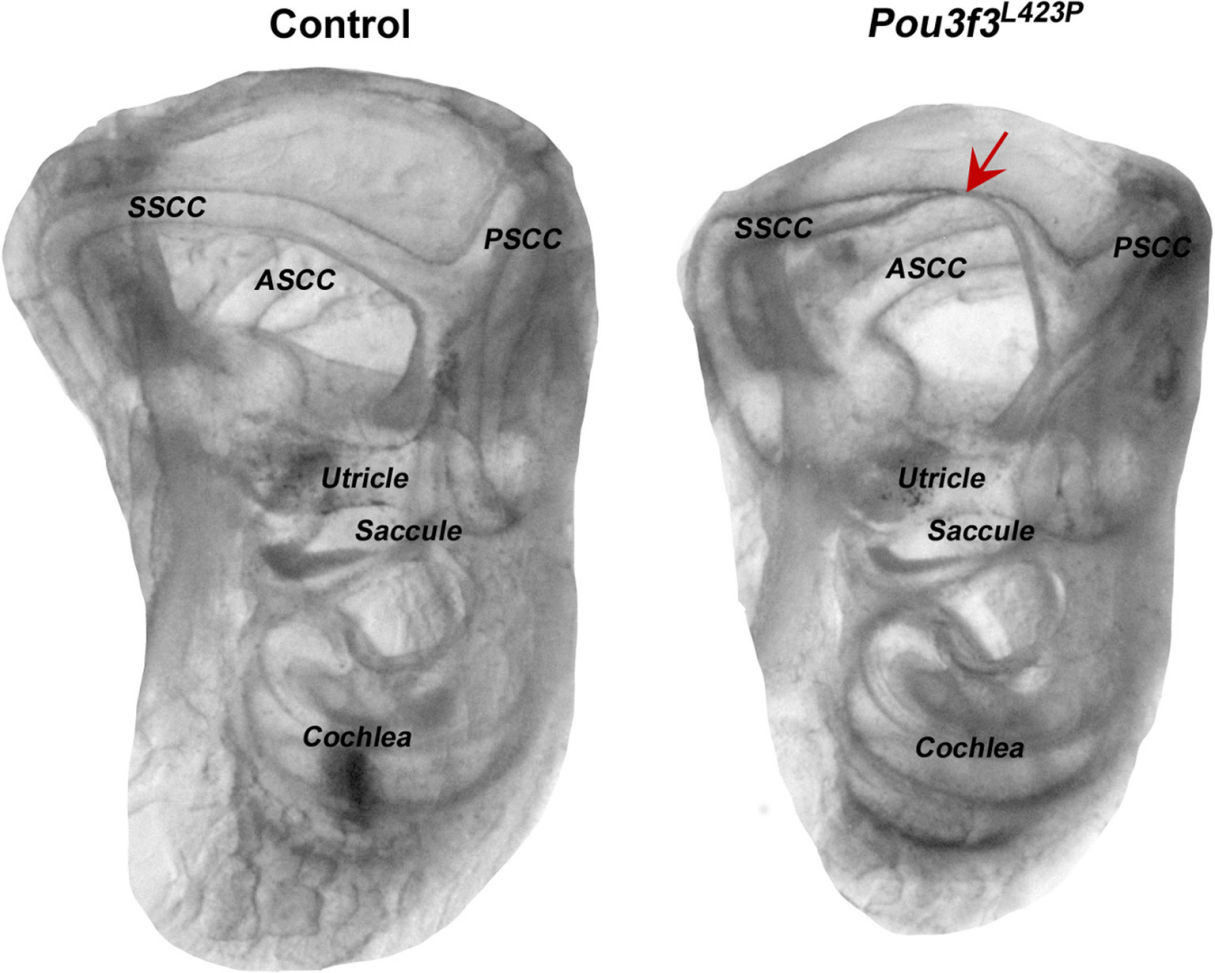
Morphological analysis of the inner ears of line *Pou3f3*^*L423P*^. Morphological analysis of the inner ears using the whole mount clearing method showed abnormal superior semi-circular canal formation in the homozygous mutants of line *Pou3f3*^*L423P*^. Left picture shows a control inner ear with normal semi-circular canals. Right picture shows the smaller inner ear of a *Pou3f3*^*L423P*^ homozygous mutant animal with abnormal superior semi-circular canal formation. Red arrow shows the constriction of the superior semi-circular canal. SSCC: superior semi-circular canal, PSCC: posterior semi-circular canal, ASCC: anterior semi-circular canal.

Morphological analysis of the inner ears using the whole mount clearing method (n = 6 per genotype) showed abnormal superior semi-circular canal formation of the vestibular organ of the mutants (Fig 3). The canals appear intact on both sides but the thickness and the curvature of the superior semi-circular canal is impaired. The side of the superior semi-circular canal that meets the common crus presents an irregular curvature of the canal that might be responsible for the balance deficits in the mutants.

The prepulse inhibition (PPI) of the acoustic startle reflex test assessed the sensorimotor gating. Acoustic startle reactivity and prepulse inhibition were significantly decreased in mutant mice at all sound pressure levels, i.e. startle amplitude at 70, 80, 85, 90, 100, 110 and 120 dB, and prepulse inhibition at 4, 8 and 12 dB prepulse sound pressure level above background (*P* < 0.001 for all). The clear reduction of the acoustic startle amplitude suggested either impaired hearing ability or impaired neuromuscular recruitment. By measuring the auditory brain stem response (ABR), hearing sensitivity was assessed in a subset of mice (7 male and 3 female mutants as well as 9 male and 5 female controls; 16 weeks of age) by recording the minimum sound pressure required to elicit a physiological response to different auditory stimuli. There were significant (*P* ≤ 0.001) differences at all tone frequencies (6, 12, 18, 24 and 30 kHz) as well as for the broadband click. Sound pressure thresholds were clearly increased in both sexes, indicating decreased hearing sensitivity.

### Nociception

Analysis of nociception was carried out in 10-week-old mice by using the hot plate assay at 52°C for the detection of altered pain response. The reactions due to thermal pain by shaking and licking of the hind paws were measured in the mutants and controls (latency in seconds for shacking of hind paws: 15.4 ± 7.5 vs. 14.3 ± 4.1 in males, 18.8 ± 6.7 vs. 12.5 ± 4.3 in females (*P* = 0.02); latency in seconds for licking of hind paws: 24.6 ± 7.4 vs. 23.8 ± 5.7 in males, 28.0 ± 2.9 vs. 21.0 ± 5.7 in females (*P* = 0.01); n = 13–16 per genotype and sex). Thus, the female mutants showed a significant hypoalgesia.

### Clinical Chemistry and Hematology Analysis

Line *Pou3f3*^*L423P*^ was established based on increased plasma urea levels in the homozygous mutant animals. Clinical chemical analysis of blood plasma at the age of 15 weeks revealed significantly increased values for creatinine, urea, chloride, potassium as well as alkaline phosphatase, aspartate aminotransferase and lactate dehydrogenase activity, and decreased values for glucose, albumin, total protein, cholesterol and triglycerides in the mutants (Table 4). In view of the lower body weight and increased locomotor activity, the plasma parameters with the decreased values might indicate a state of low energy stores in the mutants. In the hematology analysis, mutants showed decreased values for red blood cell count, hemoglobin, hematocrit, mean corpuscular volume and mean corpuscular hemoglobin. Thus, they showed erythropenic anemia with a trend towards microcytosis. This might be a secondary effect of the azotemia. In addition, platelets were also decreased, whereas white blood cells were increased in the mutants (Table 5).

**Table 4:** Clinical chemical analysis of line *Pou3f3*^*L423P*^.

	Males		Females		Genotype: *P*-value
Parameter	Homozygous mutant	Wild-type	Homozygous mutant	Wild-type	
Na^+^ (mmol/l)	153 ± 1	153 ± 1	151 ± 3	150 ± 4	
K^+^ (mmol/l)	5.1 ± 0.4	4.6 ± 0.3	4.7 ± 0.3	3.9 ± 0.3	c
Ca^2+^ (mmol/l)	2.4 ± 0.1	2.5 ± 0.1	2.5 ± 0.1	2.4 ± 0.1	
Cl^-^ (mmol/l)	109 ± 1	108 ± 2	112 ± 3	110 ± 3	b
Fe (μmol/l)	38.0 ± 3.4	36.8 ± 3.8	40.5 ± 8.8	37.6 ± 3.8	
P_i_ (mmol/l)	1.5 ± 0.3	1.5 ± 0.2	1.7 ± 0.3	2 ± 0.4	
Total protein (g/l)	53.2 ± 1.4	56.1 ± 1.8	52.7 ± 1.3	53.5 ± 2.4	c
Albumin (g/l)	29.7 ± 3.4	31.5 ± 1	30.5 ± 2.5	32 ± 1.6	b
Creatinine (μmol/l)	16.5 ± 1.3	15.9 ± 1.0	16.5 ± 2.8	11.1 ± 2.4	c
Urea (mmol/l)	20.8 ± 1.8	10.7 ± 0.9	18.7 ± 2.1	8.7 ± 1.3	c
Cholesterol (mmol/l)	3.5 ± 0.2	4.3 ± 0.4	3.1 ± 0.2	3.3 ± 0.4	c
Triglycerides (mmol/l)	2.2 ± 0.6	3.4 ± 0.8	2.1 ± 0.6	3.0 ± 1.0	c
ALT (U/l)	45 ± 50	33 ± 7	27 ± 5	28 ± 6	
AST (U/l)	72 ± 30	51 ± 8	72 ± 22	48 ± 7	c
AP (U/l)	132 ± 14	111 ± 9	148 ± 25	132 ± 14	c
α-Amylase (U/l)	717 ± 86	726 ± 55	694 ± 59	623 ± 59	
Glucose (mmol/l)	9.4 ± 1.8	10.6 ± 2.2	10.4 ± 2.0	11.5 ± 1.8	a
LDH (U/l)	250 ± 104	178 ± 38	239 ± 53	181 ± 39	c

Creatinine, plasma creatinine analyzed by the enzymatic method; ALT, alanine aminotransferase; AST, aspartate aminotransferase; AP, alkaline phosphatase; LDH, lactate dehydrogenase.

15-week-old mice were tested. No. per genotype and sex: n = 13–16. Data are presented as mean ± standard deviation. Significance vs. wild-type controls: a, *P* < 0.05; b, *P* < 0.01; c, *P* < 0.001.

**Table 5:** Hematological analysis of line *Pou3f3*^*L423P*^.

	Males		Females		Genotype: *P*-value
Parameter	Homozygous mutant	Wild-type	Homozygous mutant	Wild-type	
WBC (10^3^/μl)	8.7 ± 1.5	7.4 ± 1.3	8.7 ± 2.2	7.1 ± 1.4	b
RBC (10^6^/μl)	8.9 ± 0.3	9.5 ± 0.6	9.2 ± 0.3	9.6 ± 0.3	c
PLT (10^3^/μl)	972 ± 74	1093 ± 107	1006 ± 111	1168 ± 93	c
HGB (g/dl)	14.5 ± 0.5	15.6 ± 0.9	15.1 ± 0.5	16.0 ± 0.4	c
HCT (%)	50.2 ± 1.7	54.0 ± 3.3	52.5 ± 1.5	55.6 ± 1.5	c
MCV (fl)	56.2 ± 0.4	56.9 ± 0.7	57.0 ± 0.6	57.8 ± 0.7	c
MCH (pg)	16.2 ± 0.3	16.5 ± 0.4	16.4 ± 0.4	16.6 ± 0.2	a
MCHC (g/dl)	28.8 ± 0.4	28.9 ± 0.6	28.8 ± 0.6	28.8 ± 0.4	

WBC, white blood cell count; RBC, red blood cell count; PLT, platelet count; HGB, hemoglobin; HCT, hematocrit; MCV, mean corpuscular volume; MCH, mean corpuscular hemoglobin; MCHC, mean corpuscular hemoglobin concentration.

15-week-old mice were tested. No. per genotype and sex: n = 13–16. Data are presented as mean ± standard deviation. Significance vs. wild-type controls: a, *P* < 0.05; b, *P* < 0.01; c, *P* < 0.001.

Clinical chemical analysis of the plasma parameters of an additional group of homozygous mutant and wild-type control mice (n = 5–10 per genotype and sex) not transferred to the German Mouse Clinic at the age of 12–14 weeks confirmed the results of the GMC analysis at the age of 15 weeks. Moreover, heterozygous mutants (17 males, 8 females) were also included in this analysis and showed no significant differences compared to the wild-type controls (data not shown). In addition, metabolic cage analysis was carried out in these mice at the age of 14–15 weeks (n = 4–10 per genotype and sex). The mutant mice harbored a lower body weight and showed an increased urine volume in the males as well as decreased urinary uric acid levels indicating kidney dysfunction, also together with the other parameters showing altered urine levels by tendency in both sexes when compared to the controls (Table 6).

**Table 6:** Urine analysis of line *Pou3f3*^*L423P*^ (in an additional group of mice not transferred to the German Mouse Clinic).

	Males		Females	
Parameter	Homozygous mutant	Controls	Homozygous mutant	Controls
Body weight (g)	20.4 ± 1.4 ^c^	26.3 ± 3.6	18.2 ± 2.2 ^b^	23.3 ± 2.7
Water *ad libitum*				
Water intake (ml/day)	5.9 ± 3.0	4.3 ± 0.7	5.5 ± 1.7	4.9 ± 2.3
Food intake (g/day)	3.5 ± 1.2	3.9 ± 0.6	3.6 ± 0.7	4.2 ± 0.7
Urine volume (ml/day)	1.5 ± 0.7 ^a^	0.9 ± 0.3	1.6 ± 0.5	1.0 ± 0.6
Na^+^ (μmol/day)	125 ± 47	169 ± 36	170 ± 69	231 ± 67
K^+^ (μmol/day)	375 ± 114	422 ± 149	505 ± 117	600 ± 232
Ca^2+^ (μmol/day)	1.5 ± 0.5	1.4 ± 0.6	3.0 ± 0.8	2.0 ± 0.9
Cl^-^ (μmol/day)	217 ± 78	274 ± 73	315 ± 112	395 ± 118
Mg^2+^ (μmol/day)	9 ± 7	15 ± 9	23 ± 13	22 ± 9
P_i_ (μmol/day)	50 ± 54	63 ± 47	77 ± 71	99 ± 59
Creatinine-J (μmol/day)	3.5 ± 1.0	4.0 ± 1.0	3.8 ± 1.1	4.9 ± 1.3
Creatinine-E (μmol/day)	2.1 ± 0.5	2.5 ± 0.6	2.5 ± 0.7	3.1 ± 0.9
Urea (mmol/day)	1.2 ± 0.3	1.3 ± 0.4	1.6 ± 0.4	1.8 ± 0.6
Uric acid (nmol/day)	329 ± 94 ^b^	587 ± 154	522 ± 84 ^b^	977 ± 208
Glucose (μmol/day)	1.5 ± 1.1	2.1 ± 1.1	5.0 ± 2.5 ^a^	3.1 ± 0.8
Total protein (mg/day)	5.0 ± 1.7	7.3 ± 3.7	0.9 ± 0.3	2.9 ± 2.1
Albumin (nmol/day)	1.4 ± 0.3	1.9 ± 0.6	2.0 ± 0.2	2.2 ± 0.7
Deprivation of water for 24 h				
Loss of body weight (%)	13.2 ± 2.2 ^b^	9.6 ± 1.4	13.4 ± 1.2 ^c^	9.7 ± 1.3
Food intake (g/day)	1.2 ± 0.4 ^c^	2.3 ± 0.3	1.8 ± 0.2 ^b^	2.3 ± 0.3
Urine volume (ml/day)	0.6 ± 0.3	0.5 ± 0.2	0.7 ± 0.1	0.5 ± 0.2

Creatinine-J, creatinine analyzed by Jaffe’s kinetic method; Creatinine-E, creatinine analyzed by the enzymatic method.

The analysis was done under basal conditions and after deprivation of drinking water for 24 h in metabolic cages. 14-15-week-old mice were tested. Heterozygous mutant and wild-type littermates were used as controls. No. per group and sex: n = 4–10. Data are presented as mean ± standard deviation. Student’s *t*-test vs. controls: ^a^ *P* < 0.05, ^b^ *P* < 0.01, ^c^ *P* < 0.001.

In addition, an intraperitoneal glucose tolerance test (IpGTT) was carried out at the age of 12 weeks after food withdrawal overnight (n = 13–16 per genotype and sex). Relative body mass loss induced by overnight food withdrawal showed no clear genotype effect. In the food-deprived condition, mutants showed a higher glucose clearance from 30 min after the start of the test compared to the wild-type controls (basal glucose level (mmol/l): 6.1 ± 0.6 vs. 6.2 ± 0.6 in males, 4.9 ± 0.5 vs. 5.0 ± 0.7 in females (*P* = 0.6); area under the curve (AUC) for the first 30 min: 321 ± 50 vs. 290 ± 64 in males, 219 ± 42 vs. 245 ± 54 in females (*P* = 0.9); AUC for the remaining 90 min: 447 ± 148 vs. 911 ± 197 in males, 261 ± 73 vs. 422 ± 208 in females (*P* < 0.001)).

### Dysmorphology, Bone and Cartilage

*Pou3f3*^*L423P*^ homozygous mutant mice were tested at the age of 10 (morphological analysis), 11 (click box test) and 19 (dual energy X-ray absorption (DXA) and X-ray analysis) weeks. The systematic morphological investigation via visual inspection (overall body shape, eye, coat, skin, vibrissae, extremities including limbs, digits and tail, teeth, ears, musculature, skeleton, neurology/behavior, respiratory system, reproductive system, other aberrant phenotype) found no genotype-specific differences. Analysis of the hearing ability in 11-week-old mice was examined by the click box test using a sound of 20 kHz, and the reaction of the animals (n = 13–16 per genotype and sex) was classified into six categories. We observed a decreased hearing reaction in the mutants by trend (*P* = 0.1 for males, *P* = 0.3 for females).

DXA (dual energy X-ray absorption) analysis detected significantly decreased values for bone mineral density (BMD) in male mutants, bone mineral content (BMC) in all mutants, and bone content in male mutants compared to controls. This might be a secondary effect of the renal dysfunction leading to the impaired electrolyte balance in the mutant mice which was revealed in the clinical chemistry analysis. Concurrently body length, body weight, and fat mass were significantly decreased in mutants (Table 7). X-ray analysis of the skeleton (skull shape, mandibles, maxilla, teeth, orbit, number of vertebrae, vertebrae shape, number of ribs, rib shape, scapula, clavicle, pelvis, femur diameter, femur shape, tibia, fibula, humerus, ulna, radius, number of digits, completeness of digits, joints) found no genotype-specific differences between mutants and wild-type controls.

**Table 7:** Dual energy X-ray absorption (DXA) analysis of bone- and weight-related parameters in line *Pou3f3*^*L423P*^.

	Males		Females	
Parameter	Homozygous mutant	Wild-type	Homozygous mutant	Wild-type
BMD (mg/cm^2^)	54 ± 6 ^b^	63 ± 5	54 ± 5	61 ± 6
BMC (mg)	548 ± 79 ^c^	883 ± 246	522 ± 196 ^a^	804 ± 249
Bone content (%)	2.0 ± 0.3 ^a^	2.4 ± 0.6	2.0 ± 0.4	2.3 ± 0.4
Body length (cm)	9.9 ± 0.2 ^b^	10.3 ± 0.2	9.9 ± 0.4 ^a^	10.3 ± 0.4
Body weight (g)	28.1 ± 2.7 ^c^	36.3 ± 3.3	25.2 ± 4.8 ^c^	33.8 ± 6.3
Fat mass (g)	5.8 ± 1.8 ^c^	15.1 ± 5.5	6.6 ± 4.7 ^b^	14.7 ± 7.3
Lean mass (g)	16.0 ± 1.2	14.0 ± 4.0	12.9 ± 1.3	12.7 ± 3.2

BMD, bone mineral density; BMC, bone mineral content.

Analysis was done at the age of 19 weeks. No. per genotype and sex: n = 8–16. Data are presented as mean ± standard deviation. Significance vs. wild-type controls: ^a^ *P* < 0.05, ^b^ *P* < 0.01, ^c^ *P* < 0.001.

### Energy Metabolism

Analysis of the energy metabolism was done on 11-week-old mice under *ad libitum* conditions. Indirect calorimetry revealed that mutants were considerably underweight and showed a marked reduction in rectal body temperature. The other effects on energy turnover were confounded by the difference in body weight. When adjusted for body weight, significant genotype effects on residual mean, minimum and maximum oxygen consumption were observed especially in the female mutants showing a slight hypometabolism. Despite lower metabolic rate, we observed a mild increase of locomotor activity but not rearing behavior. We could not detect a significant difference in the respiratory exchange ratio (RER) but the relation between the levels of oxygen consumption and metabolic fuel utilization indicated that metabolic flexibility may be impaired in mutant mice. At higher levels of VO_2_, the controls showed a wider range of respiratory exchange ratios than the mutant mice (not shown). In the determination of body composition by time domain nuclear magnetic resonance (TD-NMR), mutant mice also showed a significantly lower body weight than control mice. The statistical analysis did not reveal significant genotype related main effects on body fat content or lean mass when adjusted for body mass but it was evident that the relation between body weight and fat mass respectively lean mass was different between control and mutant mice (Table 8).

**Table 8:** Analysis of energy metabolism in line *Pou3f3*^*L423P*^.

		Males		Females		Genotype: *P*-value
Test	Parameter	Homozygous mutant	Wild-type	Homozygous mutant	Wild-type	
Indirect calorimetry	Body weight (g)	24.3 ± 1.3	30.7 ± 2.5	20.9 ± 2.1	28.4 ± 3.3	c
	Body temperature (°C)	35.7 ± 0.7	36.2 ± 0.4	36.4 ± 0.3	36.8 ± 0.3	b
	Food intake (g)	2.8 ± 0.3	3.2 ± 0.8	2.8 ± 0.4	3.6 ± 0.6	
	Average VO_2_ consumption (ml/h animal)	85 ± 4	95 ± 6	81 ± 6	99 ± 7	a
	Average RER (VCO_2_/VO_2_)	0.89 ± 0.01	0.90 ± 0.04	0.90 ± 0.02	0.92 ± 0.03	
	Adjusted mean MR (mW)	507 ± 21	508 ± 21	515 ± 23	556 ± 50	0.05
	Adjusted RMR (mW)	375 ± 17	383 ± 23	392 ± 11	492 ± 24	c
	Average distance (cm/20 min)	843 ± 213	658 ± 86	958 ± 400	810 ± 298	0.06
	Average rearings (counts/20 min)	144 ± 59	119 ± 20	144 ± 71	149 ± 69	
TD-NMR	Body weight (g)	24.8 ± 1.6	30.5 ± 2.6	20.1 ± 2.8	27.4 ± 3.2	c
	Fat mass (g)	5.4 ± 0.6	7.8 ± 1.4	4.9 ± 1.1	8.0 ± 1.9	
	Lean mass (g)	15.8 ± 1	18.9 ± 1.3	12.2 ± 1.5	16.1 ± 1.5	0.06

RER, respiratory exchange ratio; adjusted mean MR, metabolic rate (MR) was adjusted for body mass by subtracting residual MR from modeled mean expected MR; adjusted RMR, adjusted resting metabolic rate (i.e. lowest MR during the measurement); TD-NMR, time domain nuclear magnetic resonance.

Analysis was done on 11-week-old mice under *ad libitum* conditions. No. per genotype and sex: n = 9–16. Substrate utilization rates during indirect calorimetry were calculated as described [27]. Data are presented as mean ± standard deviation. Significance vs. wild-type controls: a, *P* < 0.05; b, *P* < 0.01; c, *P* < 0.001.

### Immunology

The mice were tested at 15 weeks of age for leukocyte populations in peripheral blood and immunoglobulin levels (IgM, IgG1, IgG2a, IgG2b, IgG3, and IgA) as well as rheumatoid factor and anti-DNA antibodies in blood plasma. Flow cytometry observed a higher frequency of granulocytes as well as a lower frequency of CD8+ T cells resulting in the tendency of a higher CD4:CD8 ratio in female mutants. Furthermore, we found higher frequencies of CD44 expressing cells within both (CD4+ and CD8+) T cell clusters. Only in female mutants we found lower frequencies of B cells and NK cells and an increased frequency of CD25 expressing cells within the CD4+ T cell cluster (Table 9). The frequency of granulocytes in blood showed changes which have been described for various pathological conditions (e.g. tumor development, infection, stress). Thus, the increased frequency of granulocytes might be a major factor for the changes in the main leukocyte populations in the mutants. The T cells showed differences in the frequencies of CD44 expressing cells. The high-surface expression of the CD44 is assigned to activated T cells; alternatively, these cells might represent cells that arise via physiological homeostatic proliferation [15].

**Table 9:** Immunology analysis of line *Pou3f3*^*L423P*^.

	Males		Females		Genotype: *P*-value
Parameter	Homozygous mutant	Wild-type	Homozygous mutant	Wild-type	
CD45+/T cells	33.9 ± 4.4	36.4 ± 3.2	36.6 ± 4.4	39.2 ± 5.2	a
CD45+/CD3+CD4+	22.0 ± 3.1	23.5 ± 2.5	23.5 ± 2.8	25.1 ± 4.0	0.07
CD45+/CD3+CD8+	8.9 ± 1.1	9.7 ± 1.0	10.2 ± 1.6	11.4 ± 1.2	b
CD45+/B cells	28.7 ± 3.5	28.7 ± 2.7	28.3 ± 5.0	33.1 ± 5.8	a
CD45+/CD5-NK+	6.3 ± 1.4	6.9 ± 1.6	4.4 ± 1.0	5.7 ± 1.2	b
CD45+/CD11b+Gr1+	28.6 ± 6.1	25.6 ± 3.7	28.0 ± 6.1	17.5 ± 5.7	c
CD45+/NK-Gr1-CD11b+	2.2 ± 0.9	2.7 ± 0.6	2.3 ± 0.7	2.6 ± 1.4	
CD45+/CD3+γδTCR+	0.99 ± 0.2	0.94 ± 0.3	0.99 ± 0.3	0.98 ± 0.3	
CD45+/CD5+NK+	0.06 ± 0.05	0.05 ± 0.03	0.02 ± 0.01	0.02 ± 0.02	
CD45+/CD3+CD4+CD25+	0.89 ± 0.17	0.89 ± 0.14	1.03 ± 0.25	0.94 ± 0.11	
CD3+CD4+/CD25+	4.1 ± 0.6	3.8 ± 0.5	4.4 ± 1.2	3.8 ± 0.4	a
CD3+CD4+/CD62L+	90.0 ± 3.9	91.3 ± 2.6	85.5 ± 8.5	86.2 ± 5.3	
CD3+CD4+/CD44+	2.5 ± 0.4	2.0 ± 0.3	3.1 ± 0.7	2.3 ± 0.5	c
CD3+CD8+/CD62L+	97.6 ± 1.0	97.5 ± 0.9	97.2 ± 1.7	97.4 ± 1.1	
CD3+CD8+/CD44+	3.5 ± 0.9	3.3 ± 0.9	2.3 ± 0.8	1.6 ± 0.8	a
B cells/IgD+	89.3 ± 2.8	88.1 ± 2.8	87.4 ± 2.9	86.6 ± 3.2	
B cells/CD5+	5.3 ± 1.7	5.3 ± 1.0	5.2 ± 2.0	5.1 ± 1.5	
B cells/ B220+MHCclassII+	97.9 ± 1.0	98.2 ± 0.6	93.6 ± 5.1	95.4 ± 3.1	
NK+/CD11b+	9.5 ± 6.3	12.9 ± 6.5	3.4 ± 1.9	3.0 ± 2.4	
CD3+ rest	2.9 ± 0.5	3.1 ± 0.4	2.8 ± 0.7	2.7 ± 0.5	
ratio CD4: CD8	2.5 ± 0.1	2.4 ± 0.2	2.3 ± 0.2	2.2 ± 0.2	
ratio granulocytes: (T cells + B cells)	0.46 ± 0.13	0.40 ± 0.07	0.44 ± 0.13	0.25 ± 0.10	c
ratio T cells: B cells	1.20 ± 0.24	1.28 ± 0.18	1.33 ± 0.29	1.22 ± 0.27	

Data are frequencies of main leukocyte subpopulations in peripheral blood (%) measured by flow cytometry.

15-week-old mice were tested. No. per genotype and sex: n = 13–16. Data are presented as mean ± standard deviation. Significance vs. wild-type controls: a, *P* < 0.05; b, *P* < 0.01; c, *P* < 0.001.

For the immunoglobulin levels, slightly lower levels of immunoglobulins were found in male mutants compared to controls. Especially, the levels of IgG3 were significantly lower (data not shown).

In total, we found multiple changes in the frequencies of leukocyte subset proportions in peripheral blood. Most of the observed changes were subtle, however, the accumulation of the immunological findings may suggest a primary or secondary influence of the mutation on the immune system.

### Molecular Phenotyping

Based on the results of the analysis in the German Mouse Clinic as well as on the published results of *Pou3f3* knockout mice [7,8,16], the pathogenic effects of the *Pou3f3* mutation were evaluated in brain, kidney and spleen by genome-wide transcriptome profiling analysis using Illumina MouseRef8 v2.0 Expression Bead Chips. Samples of the three selected organs of four mutant males were compared to four wild-type males as controls at the age of 24 weeks (in total 23 experiments as the RNA isolation of one control kidney sample failed).

Statistical analysis of the gene expression patterns [17,18] in the brain identified 104 significantly regulated genes in mutants vs. wild-type controls (false discovery rate (FDR) = 0.6%, fold change > 1.4). The range of the mean log_2_ ratios was 1.6 to 2.0 for the 7 up-regulated genes as well as -2.0 to -1.4 for 96 down-regulated genes and -5.0 for *Tshb* (thyroid stimulating hormone, beta subunit) as additionally down-regulated gene. The genes are functionally associated with movement disorders, Huntington’s disease, necrosis and RNA transcription.

Statistical analysis of the gene expression patterns in the kidney identified 292 significantly regulated genes in mutants vs. wild-type controls (FDR = 1.5%, fold change > 1.4). The range of the mean log_2_ ratios was 2.0 to 3.4 for the 22 up-regulated genes and -22.8 to -1.7 for the 270 down-regulated genes (with -22.8 to -5.0 for the genes *Sycn*, *Wfdc15b*, *Cldn10*, *Shd*, *Higd1c*, *Gpx6*, *Popdc3*, *Slc6a12*, and *Mettl7a2*). The genes are functionally associated with concentration of metal ions, lipids, proteins and steroids as well as renal impairment and failure.

Statistical analysis of gene expression patterns in the spleen identified 124 significantly regulated genes (FDR = 0%, fold change > 1.4), all of them were down-regulated. The range of the mean log_2_ ratios was -3.9 to -1.5. The genes are functionally associated with inflammatory response including proliferation/migration/movement of leukocytes, phagocytes, granulocytes and lymphocytes.

The genes *Asah1*, *Cnot2*, *Ctps2*, *Ddx5*, *Lgsn*, *Lypla1*, *Mtmr2* and *Rcan1* were down-regulated in all three organs examined, the genes *Armc 8*, *Atp6ap2*, *Dars*, *Hsd11b1*, *Sh3gl2*, *Snx14*, *Tmem49*, *Wdr37* and *Yme1l1* were down-regulated in both brain and kidney, the gene *Sec22a* was down-regulated in both brain and spleen, and the genes *Cav2*, *Cd9*, *Cyp2b10*, *Mal*, *Pira11*, *Stap1*, *Thbs1* and *Vps41* were down-regulated in both kidney and spleen. The genes showed a range of their mean log_2_ ratios between -2.8 to -1.4. Despite this overlap of differential gene expression in the organs, necrosis was found as the only gene-specific functional annotation over-represented in brain and kidney. The other enriched terms were associated with organs-specific functions.

## Discussion

The recessive mutant line HST011 was established on the C3H inbred genetic background based on increased plasma urea levels, and the causative mutation was identified as *Pou3f3*^*L423P*^ thereby affecting the highly conserved homeobox domain of the protein. *Pou3f3* is a transcription factor that is expressed in the central nervous system [7,19], the inner ear and some other organs like the kidney [8] during embryonic development. POU3F3 acts synergistically with SOX11 and SOX4 (http://www.uniprot.org).

Two complete POU3F3 knockout mutant mouse lines have been described to date. The first knockout mouse line was established by replacing the gene sequences which encode the first 476 amino acids with a PGK-neo cassette via homologous recombination on an unspecified genetic background [7]. The second knockout line was also established by using a PGK-neo cassette for replacing 1.2 kb of the coding region. Functional studies were performed on the mixed genetic background of 129S4/SvJae × C57BL/6J mice [8]. Heterozygous mutant mice were reported to be phenotypically normal in both knockout lines. Our heterozygous mutant mice also showed a grossly normal phenotype. *Pou3f3* homozygous knockout mice showed neonatal mortality. In contrast, *Pou3f3*^*L423P*^ homozygous mutant mice are viable and fertile; however they exhibit a reduced body weight that was about 20–25% less than that of wild-type controls.

The neonatal homozygous knockout mice showed developmental defects in the forebrain [7]. In humans, a heterozygous de novo 360 kb deletion in chromosome band 2q12.1 resulting in the loss of the genes *POU3F3* and *MRPS9* was identified in a two-year-old boy with mild dysmorphic features of the head, mild motor delay and mild intellectual disability. The haploinsufficiency of *POU3F3* was suggested to be the cause for the aberrant phenotype [20]. Eight-to-24 week-old *Pou3f3*^*L423P*^ homozygous mutant mice were phenotypically examined in the German Mouse Clinic. Our experiments observed deficits in locomotor and coordination behavior as well as in hearing ability. In the inner ear of rats, *Pou3f3* was shown to be expressed specifically in nonsensory supporting cells and mesenchymal cells of the cochlea at early postnatal stages. Neonatal *Pou3f3* homozygous knockout mice did not exhibit any abnormality in the cochlea [21]. The observed morphological alterations of the canals of the vestibular organ in *Pou3f3*^*L423P*^ homozygous mutant mice indicate that *Pou3f3* is important for the proper development of the vestibular organ. The observed hearing impairment also might be attributed to alterations in the postnatal functional development or maintenance of the inner ear. Thus, the observed deficits in behavioral and neurological parameters of *Pou3f3*^*L423P*^ homozygous mutant mice might be the consequence of the morphological alterations and neuronal disorganization in the forebrain described previously as well as of the vestibular dysfunction.

One-day-old *Pou3f3* homozygous knockout mice have been described to show a severe retardation of the normal development of Henle’s loop, the distal convoluted tubule and the macula densa. They exhibited increased plasma urea and potassium levels with renal hypoplasia, and died within 24 hours of birth because of renal failure. In the kidneys of heterozygous knockout mice, the expression of a number of genes that are specific for the thick ascending limb of Henle’s loop was reduced including *Umod*, *Nkcc*2/*Slc12a1*, *Bsnd*, *Kcnj1* and *Ptger3*. These results demonstrate the crucial role for *Pou3f3* in distal tubule formation and function in the mammalian kidney [8]. We already described the appearance of lower relative kidney weights in *Pou3f3*^*L423P*^ homozygous mutants [4]. *Pou3f3*^*L423P*^ homozygous mutant mice showed impaired renal function indicated by increased plasma levels of urea and creatinine, alterations in plasma potassium, chloride and protein levels, as well as an increased urine volume and altered renal excretion of uric acid. X-ray imaging did not reveal skeletal abnormalities, besides decreased body size and body weight, but bone mineral density was found to be decreased in homozygous mutants. The low bone mineralization may be a secondary effect due to kidney dysfunction. Altered bone mineralization was already found in previously described mouse lines showing impaired kidney function due to ENU induced mutations in the genes *Slc12a1* [22] and *Umod* [23,24]. The observation of erythropenic anemia also may be a secondary effect due to kidney dysfunction.

The analysis of energy metabolism in *Pou3f3*^*L423P*^ homozygous mutant mice indicated reduced body temperature and reduced metabolic rate compared to wild-type controls. The results of the analysis of energy metabolism were supported by the changes of clinical chemical parameters associated with fat metabolism in homozygous mutants, namely blood lipid levels and possibly also protein concentrations. A lower body fat content was already found in previously described mouse lines showing impaired kidney function due to ENU induced mutations in the genes *Slc12a1* [25] and *Umod* [23,24]. In addition, both *Umod* mutant mouse lines also showed a reduced body temperature [24,26].

The multiple subtle changes in immunological parameters may be caused by a primary or secondary effect of the mutation on the immune system. Genome-wide transcriptome profiling analysis of brain, kidney and spleen of *Pou3f3*^*L423P*^ homozygous mutants identified significantly regulated genes as compared to wild-type controls. They have to be examined in detail in further analyses. In addition, allergy, cardiovascular and steroid analyses of the German Mouse Clinic revealed no obvious differences of mutant and control mice (data not shown). Also the histological analysis (HE staining) of the organs of 24-week-old animals showed no genotype-specific differences (data not shown).

In total, as the first viable mouse line harbouring a *Pou3f3* mutation, standardized, systemic phenotypic analysis of *Pou3f3*^*L423P*^ homozygous mutants at the age of 2–6 months in the German Mouse Clinic revealed major phenotypic changes including low body weight, kidney dysfunction and secondary effects thereof as well as neurological dysfunctions.

## Supporting Information

S1 Table Time points of the phenotypic analyses for line *Pou3f3*^*L423P*^ in the German Mouse Clinic (GMC). (DOCX)
