# Supplementary material for: Generation and Standardized, Systemic Phenotypic Analysis of Pou3f3L423P Mutant Mice
Source: PLoS One. 2016 Mar 22;11(3):e0150472. doi: 10.1371/journal.pone.0150472 (PMC4803225; doi:10.1371/journal.pone.0150472)
Supplement: S1 Table — (DOCX) [file pone.0150472.s001.docx]

**S1 Table. Time points of the phenotypic analyses for line *Pou3f3^L423P^* in the German Mouse Clinic (GMC)**

| Phenotypic analysis |  | Age in weeks |
| --- | --- | --- |
| Start and end of the GMC analysis |  | 8-24 |
| Behavior | Open field | 8 |
| Neurology | Modified SHIRPA, grip strength, rotarod, acoustic startle and prepulse inhibition | 8-9 |
| Nociception | Hotplate | 10 |
| Dysmorphology | Morphological observation | 10 |
| Metabolism | Indirect calorimetry, time domain nuclear magnetic resonance | 11 |
| Clinical chemistry | Simplified intraperitoneal glucose tolerance test | 12 |
| Cardiovascular | Echocardiography | 13 (no obvious differences; not shown) |
| Eyes | Scheimpflug analysis, optical coherence tomography, eye size | 14 (not shown) |
| Clinical chemistry | Clinical chemistry, hematology | 15 |
| Immunology | FACS analysis | 15 |
| Allergy | Plasma IgE level | 15 (no obvious differences; not shown) |
| Steroids | Plasma levels of corticosterone, androstendione, and testosterone | 15 (no obvious differences; not shown) |
| Neurology | Auditory brain stem response | 16 |
| Neurology | Balance beam, beam ladder, gait, swim ability | 19 |
| Dysmorphology | Dual energy X-ray absorption, X-ray | 19 |
| Molecular phenotyping | Expression profiling | 24 |
| Pathology | Macroscopical and histological analysis | 24 (not shown) |

The GMC analyses were carried out in homozygous mutants and homozygous wild-type mice as controls.
